# Tumor infiltrating lymphocyte clusters are associated with response to immune checkpoint inhibition in *BRAF V600*^*E/K*^ mutated malignant melanomas

**DOI:** 10.1038/s41598-021-81330-4

**Published:** 2021-01-19

**Authors:** Sebastian Klein, Cornelia Mauch, Klaus Brinker, Ka-Won Noh, Sonja Knez, Reinhard Büttner, Alexander Quaas, Doris Helbig

**Affiliations:** 1 Institute of Pathology, University Hospital Cologne, Cologne, Germany; 2 Department of Dermatology, University Hospital Cologne, Kerpener Strasse 62, 50937 Cologne, Germany; 3 Hamm-Lippstadt University of Applied Sciences, Hamm, Germany; 4 Present Address: Gerhard-Domagk-Institute of Pathology, University Hospital Münster, Albert-Schweitzer-Campus 1, Gebäude D17, Münster, Germany

**Keywords:** Cancer, Skin cancer, Tumour biomarkers, Computational biology and bioinformatics, Genetics, Biomarkers, Oncology

## Abstract

Patients with metastasized malignant melanomas (MM) are regularly treated with immune checkpoint inhibitors (CPI). Within our study, we evaluated the predictive value of tumor infiltrating lymphocyte (TIL) clusters in primary MM and its association to molecular subtypes to predict response to CPI treatment. A cohort of 90 MM patients who received CPI treatment were collected from a single center, as well as a validation cohort of 351 patients from the TCGA database (SKCM) who received standard of care. A deep-convolutional-neural network (U-Net) was trained to detect viable tumor areas on H&E whole-slide-images, following a quantitative detection of TILs with help of a separate additional neural network. The number of TIL clusters was associated with response to CPI in 90 MM patients (AUC = 0.6), even more pronounced within the sub-cohort of *BRAF V600*^*E/K*^-mutated MM patients (AUC = 0.7, n = 32). Interestingly, the TIL clusters in *NRAS*-mutated as well as wildtype MM (*BRAF*-wt, *NRAS*-wt) tumors, did not demonstrate a predictive value of CPI response (AUC = 0.5, n = 25). Moreover, PD-L1 expression had a limited predictive value within our cohort. In parallel, within an independent cohort of MM patients (TCGA, n = 351), the number of TIL clusters was associated with improved survival in *BRAF V600*^*E/K*^ mutated MM (*p* < 0.0001, n = 164) but neither in *NRAS*-mutated (55.7 months vs. 63.0 months, respectively, *p* = 0.590, n = 85) nor *BRAF/NRAS*-wildtype MM patients (52.4 months vs. 47.4 months, respectively, *p* = 0.581, n = 104). While TILs in MM have been associated with improved survival, we show—for the first time—that TIL clusters are associated with response to immunotherapy in *BRAF V600*^*E/K*^ mutated MM.

## Introduction

Immune checkpoint inhibitors (CPI) targeting the programmed death 1/programmed death-ligand 1 (PD-1/PD-L1), as well as the cytotoxic T-lymphocyte-associated antigen 4 (CTLA-4), have demonstrated promising and durable antitumor activity, and revolutionized the treatment of metastasized malignant melanoma (MM) patients^1–3^. However, a relevant fraction of patients does not benefit from monotherapy or even combined CPI regimens. Despite controversial study results, CPI treatment responses have been shown to correlate with certain quantitative markers, such as PD-L1 expression levels, as well as tumor mutational burden^2–7^.

Meanwhile, the quantitative assessment of tumor infiltrating lymphocytes (TILs) has been associated with a favorable prognosis in MM^8–10^. In addition, increased numbers of TILs were linked to response to interferon-alpha treatment in patients with advanced stage MM^11^. However, to the best of our knowledge, the prognostic value of TIL quantification from H&E images to immune checkpoint inhibition in MM remains elusive.

To explore the predictive value of quantitative assessment of TILs in malignant melanoma as well as regarding treatment response to CPI, we analyzed a cohort of 90 individuals treated with CPI at a single center, real world data and validated our findings within an independent cohort of 351 cases of malignant melanomas from the TCGA database.

## Results

### Detection of viable tumor areas and tumor infiltrating lymphocyte clusters using digitized H&E whole-slide images

**Figure 1:**
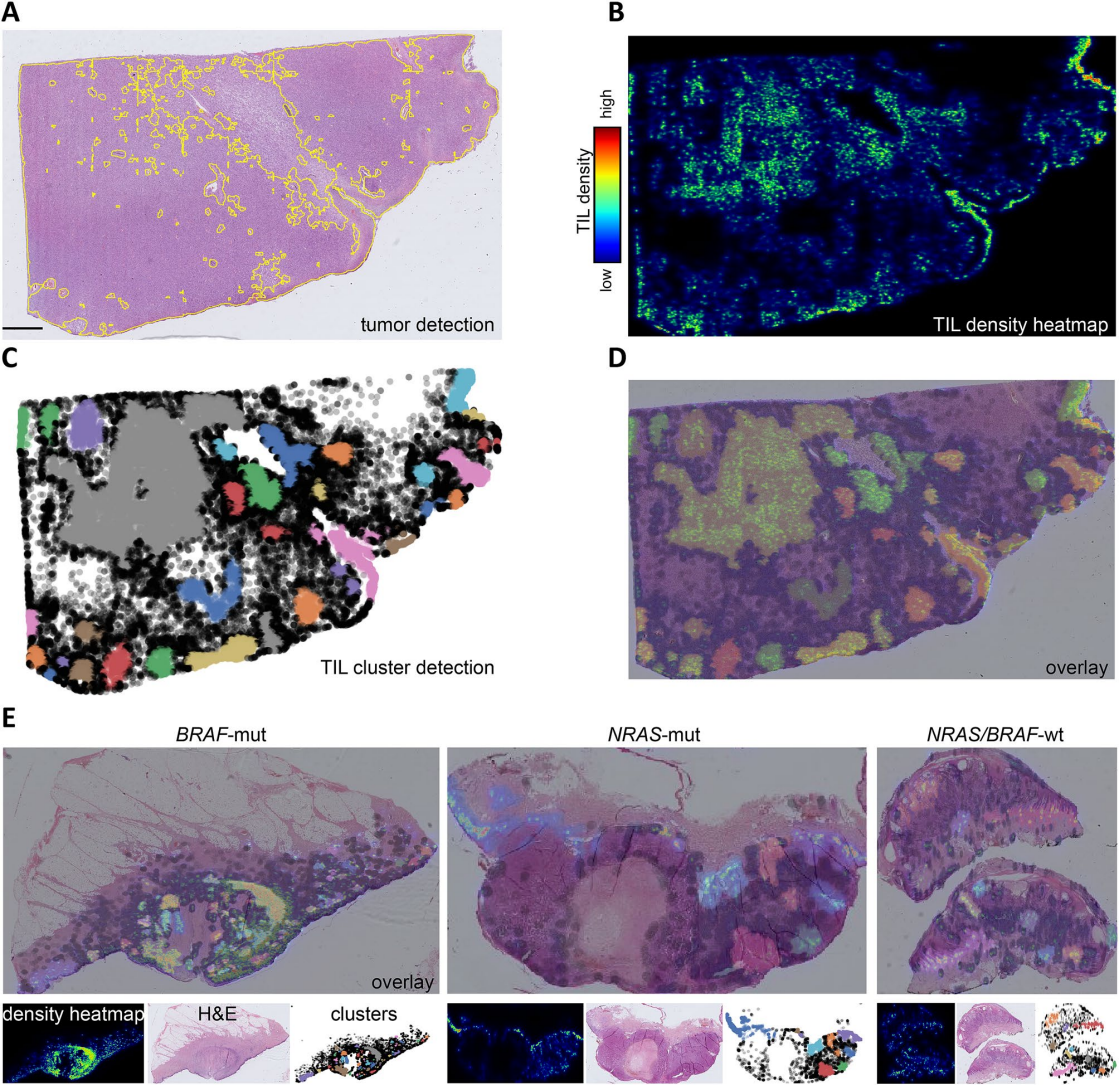
Detection of viable tumor areas and TIL clusters using regular H&E whole-slide-images of malignant melanomas. Illustration of tumor segmentation using regular H&E stains of cases of malignant melanoma, with visualizations of TIL detection and TIL clustering using single images and combined overlays. (**A**) H&E stains of one representative case of malignant melanoma with segmentation of viable tumor areas with help of a deep convolutional neural network (U-Net). The yellow line represents the segmentation of tumor area, while necrotic areas are left out. (**B**) Subsequent TIL detection within viable tumor areas and illustration of TILs using a density heatmap where red indicates high density of TILs and blue indicates low density, according to the legend. (**C**) Visualization of TIL cluster detection. Black circles indicate individual TILs and colored areas highlight distinct TIL cluster that had been assigned using HDBSCAN^12^. (**D**) Overlay of the images from panels A-C with transparent layers combining H&E image, TIL density heatmap and TIL clusters. (**E**) Panel of *BRAF*-mut (*V600*^*E/K*^) *NRAS*-mut and *BRAF/NRAS*-wildtype samples, where the overlay is shown in the upper panel and the given TIL density heatmap, TIL clustering and H&E images are shown below.

To allow a controllable and efficient detection of viable tumor areas and to avoid a bias of necrotic tumor regions with an accumulation of inflammatory cells, we trained a U-Net deep convolutional neural network for segmentation of tumor regions using digitized whole-slide images (Fig. 1A). Subsequently, we generated a neural network to detect TILs within areas of viable tumor cells (Fig. 1A).

Then, we calculated the distribution of TILs within areas of viable tumor cells by using a clustering algorithm (Fig. 1B–E)^12^. In addition to a quantitative assessment of TILs, TIL clusters would allow a qualitative assessment of TIL distribution within MM tumors.

### Predictive value of tumor infiltrating lymphocyte clusters within ***BRAF V600***^***E/K***^ mutated melanoma patients receiving CPI

**Figure 2:**
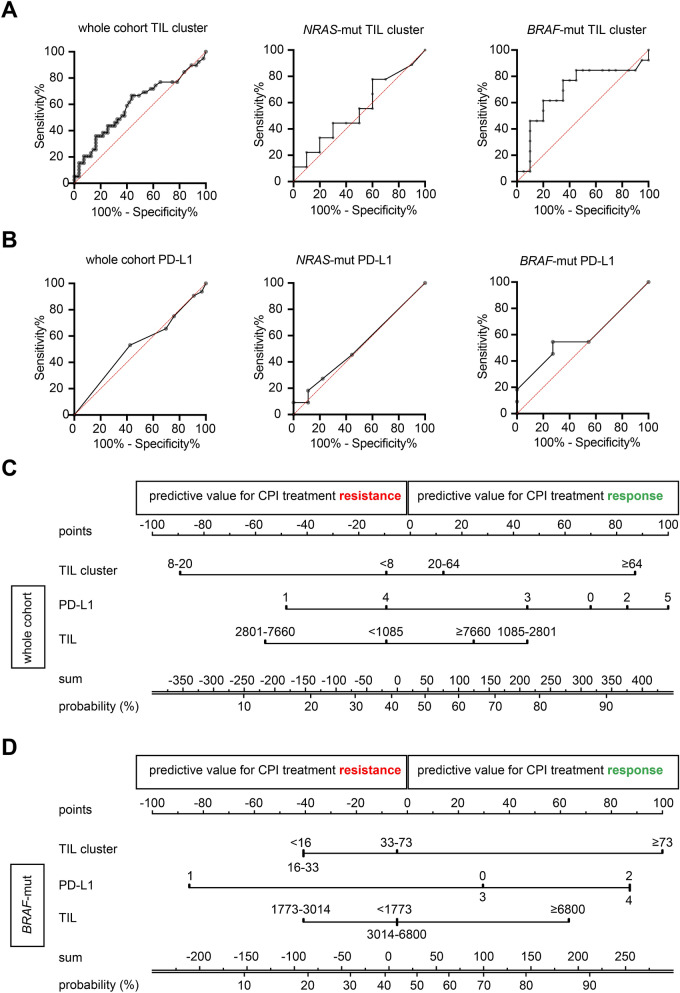
Predictive value of TIL clusters in malignant melanoma subsets. Overview of predictive value of TIL clusters according to molecular subtypes of malignant melanoma using individual ROC curves and a nomogram combining several attributes and their predictive value to predict response to CPI treatment. (**A**) Area under the curve for TIL clusters for different mutation types of MM tumors (*BRAF*-mut; *BRAF V600*^*E/K*^). (**B**) Area under the curve for PD-L1 scoring for different MM mutation subtypes. (**C**) Nomogram for the whole cohort of MM patients (n = 90). (**D**) Nomogram for the *BRAF*-mutated (*BRAF*-mut; *BRAF V600*^*E/K*^) sub cohort (n = 32) of MM patients. The positive values (points) indicate the prediction towards responses, while negative values reflect the predictive value for therapy resistance.

Having built a deep learning-based CNN for tumor segmentation, as well as a neural network for detection of TILs, we applied this method to a cohort of 90 malignant melanoma patients that received immune-checkpoint inhibition (CPI; Table 1) from a single center. Here, the overall predictive relevance to CPI response of TIL clusters was low (AUC = 0.6, n = 90; Fig. 2A). However, in 32 cases of *BRAF V600*^*E/K*^ mutated MMs, there was a higher predictive value for TIL clusters (AUC = 0.7, n = 32). Within *NRAS*-mutated MMs, there was no predictive value of TIL clusters (AUC = 0.5, n = 25).

**Table 1:** CPI treatment results.

	Ipilimumab (n = 40)	Pembrolizumab (n = 32)	Nivolumab (n = 11)	Ipilimumab + Nivolumab (n = 7)
Mean applications received ± SD (range)	3.6 ± 0.7 (1–4)	7.9 ± 5.8 (1–23)	8.7 ± 5.2 (3–17)	3.5 ± 0.8 (2–4)
**Treatment response**				
Complete response	2 (5.0%)	0	0	0
Partial response	7 (17.5%)	11 (34.4%)	5 (45.5%)	2 (28.6%)
Stable disease	3 (7.5%)	5 (15.6%)	0	0
Progress	28 (70.0%)	16 (50.0%)	6 (54.5%)	4 (57.1%)
Not evaluable	0	0	0	1 (14.3%)
Stopped treatment due to side effects	4 (10.0%)	1 (3.1%)	2 (18.2%)	1 (14.3%)

To compare the predictive value of TIL clusters to CPI treatment, TILs and PD-L1 (Fig. 2B; Table 2), we generated a nomogram for the whole cohort of MM patients (n = 90) as well as *BRAF V600*^*E/K*^ mutated MMs (n = 32). Here, TIL clusters showed an improved predictive value compared to TILs in the whole cohort as well as within *BRAF V600*^*E/K*^ mutated MMs (Fig. 2C,D). Interestingly, TIL clusters did reveal a predictive value for both response- and resistance to CPI treatment. There was a correlation between the number of TIL clusters and response to CPI treatment within *BRAF V600*^*E/K*^ mutated MM (Fig. 2D). Elevated numbers of TIL clusters (above 33) showed a higher probability of response to CPI treatment. Conversely, categorial assessment of PD-L1 showed that scoring of either 2 or 4 were associated with response, while PD-L1 scoring of 1 was linked to resistance to CPI treatment (Fig. 2D). This effect was even pronounced within the whole cohort of MM patients, as scoring of either 1 or 4 was associated with resistance to CPI treatment, compared to response to CPI treatment (scores of 3, 0 and 2; Fig. 2C).

**Table 2:** Predictive value of PD-L1 score concerning treatment outcome.

	Disease control (complete/partial response, stable disease)	Treatment non-responders (progress)	*p*
**All CPI treatments (n = 78)** **PD-L1**			
Positive	13	14	0.447
Negative	20	31	
**All anti-PD-1 treatments (nivolumab, pembrolizumab, ipilimumab + nivolumab)** **PD-L1**			
Positive	8	8	1.0
Negative	15	15	
**Ipilimumab** **PD-L1**			
Positive	5	6	0.215
Negative	5	16	
**Nivolumab** **PD-L1**			
Positive	0	1	1.0
Negative	5	5	
**Pembrolizumab** **PD-L1**			
Positive	7	5	0.654
Negative	9	9	
**Ipilimumab + Nivolumab** **PD-L1**			
Positive	1	2	1.0
Negative	1	1	

### Prognostic value of tumor-infiltrating lymphocyte clusters in molecular subtypes of malignant melanomas

**Figure 3:**
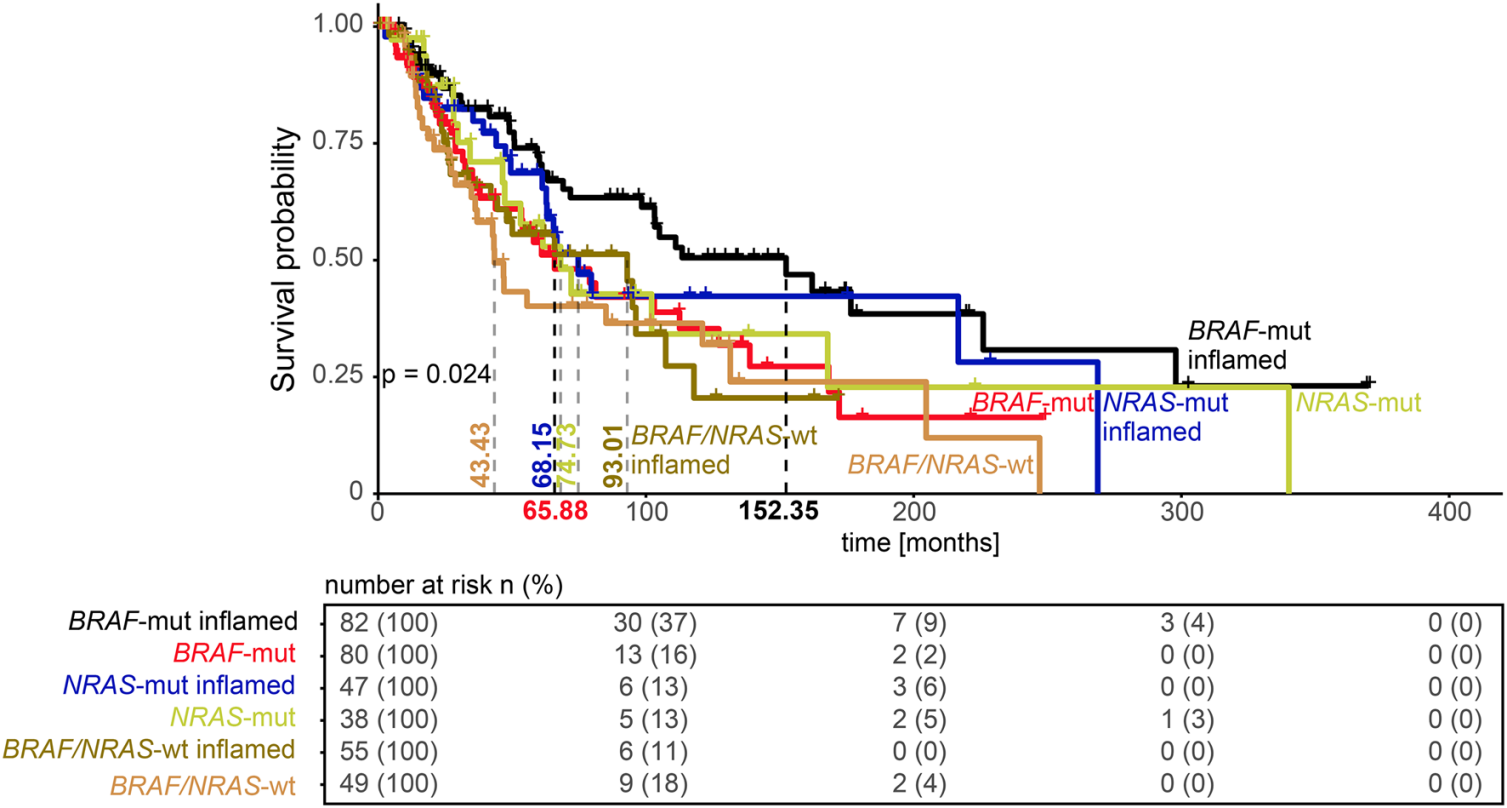
Prognostic value of TIL clusters within molecular subtypes of malignant melanomas. Kaplan–Meier curve of TCGA MM patients (n = 351) stratified for mutational subtypes (*BRAF-*mutated, *NRAS*-mutated, and *BRAF/NRAS* wildtype). Inflamed tumors are indicated according to their molecular subtype, while inflamed tumors are defined as tumors with TIL clusters above the median value as a cutoff. The median survival time for each molecular subtype and inflammatory status is provided using a vertical line and indicated in letters according to their color code. The risk table is shown below for the given subgroups.

To validate whether TIL cluster counts were associated with a favorable prognosis within molecular subtypes of MM, we analyzed an independent cohort of 351 patients from the TCGA database. Interestingly, within *BRAF V600*^*E/K*^ mutated MM tumors, the number of TIL clusters was associated with improved survival (median overall survival rate *BRAF V600*^*E/K*^ with low clusters: 48.2 vs. *BRAF V600*^*E/K*^ with high clusters: 86.9 months, *p* < 0.0001, n = 164; Fig. 3) but this effect was not seen in neither *NRAS*-mutated MM patients, (55.7 months vs. 63.0 months, respectively, *p* = 0.590, n = 85) nor wildtype MM patients (52.4 months vs. 47.4 months, respectively, *p* = 0.581, n = 104).

## Discussion

There is a clinical need to explore prognostic biomarkers for immune response to immune checkpoint inhibition in solid cancers—especially within MM—where alternative treatment strategies for molecular subtypes are available, but CPI treatment may be associated to long-term remission. Here, *BRAF V600-*mutated MM may reveal additional treatment options with BRAF and MEK inhibitors^13^.

Within our study, we highlight the predictive value of quantitative TIL cluster characterization to CPI response within *BRAF V600*-mutated MMs. Recently, there were two clinical trials performing subgroup analyses concerning response rate, progression-free and overall survival including the *BRAF* mutation status^2,3^. In a study by Larkin et al.^2^, the median overall survival among patients with *BRAF-*mutated MM was longer in the Nivolumab-plus-Ipilimumab group (more than 60.0 months; 95% CI, 50.7 to not reached), the Nivolumab group (45.5 months; 95% CI, 26.4 to not reached) and the Ipilimumab group (24.6 months; 95% CI, 17.9–31.0) compared to patients without *BRAF-*mutated MM (Nivolumab-plus-Ipilimumab group: 39.1 months; 95% CI, 27.5 to not reached, Nivolumab group: 34.4 months; 95% CI, 24.1–59.2, Ipilimumab group: 18.5 months; 95% CI, 14.1–22.7). However, the median progression-free survival was only longer among patients with *BRAF* mutations in the combination treatment group compared to patients without *BRAF* mutations (16.8 months; 95% CI, 8.3–32.0 versus 11.2 months; 95% CI, 7.0–18.1). On the other hand, Robert et al. ^3^ reported that response rates of patients receiving either Ipilimumab or Pembrolizumab were similar in patients with tumors with or without *BRAF V600*^*E/K*^ mutations.

Given the mounting evidence for TILs as a potential biomarker, our study supports to consider the feature of TIL clusters as a predictive marker for prognosis and response to CPI in MM. Assessing TILs through deep learning showed a predictive value in *BRAF V600*^*E/K*^ melanomas that received CPI treatment from a single center. These results emphasize the relevance for future studies to potentially deploy TIL clusters as a biomarker for *BRAF V600*^*E/K*^ mutated MM, where either immunotherapy or targeted therapies may be considered as treatment regimens.

## Material and methods

### Patient and tumor characteristics

90 patients with metastasized MM diagnosed at the University Hospital Cologne receiving first line CPI treatment were included in our study. All patients underwent a clinical follow-up according to the current treatment guidelines for MM. 40 patients were treated with Ipilimumab (44.4%; 3 mg/kg body weight every 3 weeks), 32 patients were treated with Pembrolizumab (35.6%; 2 mg/kg every 3 weeks) and 11 patients were treated with Nivolumab (12.2%, 3 mg/kg body weight every 2 weeks). Other 7 patients (7.8%) received Ipilimumab 3 mg/kg in combination with Nivolumab 1 mg/kg body weight every 3 weeks. Treatment response was categorized as disease control (partial/complete response or stable disease) versus disease progression according to RECIST criteria (Table 1). In general, there were more patients with disease control under Nivolumab and Pembrolizumab versus Ipilimumab (Table 1). Termination of treatment due to side effects was the highest in the Nivolumab (18.2%) and combination treatment group (14.3%; Table 1). There were no significant differences in sex, patient age at initial diagnosis or treatment initiation, tumor depth, localization, or subtype as well as mutation (*BRAF*, *NRAS* mutation or wildtype) and PD-L1 status in the different monotherapy groups. The small group of combination therapy (n = 7) contained more male patients harboring melanomas with higher Breslow index. Cases from the TCGA database received standard of care, which did not include CPI treatment at the time of sample processing.

### Molecular subtyping of MM samples

Molecular subtyping of single center cases of malignant melanomas was conducted using a targeted panel sequencing approach, covering *NRAS* (exon 2, 3, 4) and *BRAF* (exon 11, 15) among other genes as described previously^14–16^. Activating mutations for *BRAF* and *NRAS* were annotated for both TCGA and internal data^17–19^. Absence of known activating mutations in either *BRAF* or *NRAS* was considered as wildtype (*BRAF/NRAS*-wildtype; *BRAF-*wt*/NRAS*-wt), while cases with either *BRAF* or *NRAS* activating mutations were considered as *BRAF-V600*^*E/K*^* or NRAS*-mutated (*BRAF-*mut*/NRAS-*mut).

### Whole-slide-images and processing

Regular H&E stained slides, following standard protocols, were scanned using a NanoZoomer S360 (Hamamatsu Photonics) whole-slide scanning device at a 40X magnification, as well as slides from the TCGA database being scanned 20X using Leica Aperio slide scanning devices. All digitized slides were evaluated for image quality and included, if more than 90% of the tissue area was in focus. All tumors investigated were primary melanomas excised before beginning of the CPI treatment.

### Image segmentation, object detection and clustering

A U-Net was trained to detect vital tumor areas on H&E MM virtual whole slide images^20^. For this purpose, images were annotated using whole slide images by a trained pathologist. To generate a training dataset that would reflect the heterogeneity that can be observed within MM tumors, 92 image tiles (10,000 × 10,000 pixels, resized to 1000 × 1000 for further training purpose) from a total of 35 cases were used, with an image patch size of 256 pixels for the network to be processed. Training was performed using TCGA cases, as well as cases from a set of internal cases, while all cases were independent to the test set. Training was done on a NVIDIA RTX 6000 using the PyTorch framework and Adam as an optimizer. Several augmentation steps were applied, including grey-scale augmentation with a ten percent probability^21^. For TIL object detection, we followed a similar approach that has already been published^22^. To efficiently identify the number of TIL clusters, we used the HDBSCAN algorithm^12^.

### PD-L1 expression on tumor cells

A tumor cell was considered PD-L1 positive if the cell membrane was partially or completely stained, whereas cytoplasmic PD-L1 staining was not considered as a specific immune signal. The tumor proportion score was determined as previously published^23^.

### Statistical analysis

The area under the receiver-operator-curve (ROC curve) was calculated based on the overall ability of the given attributes to discriminate between patients that either responded or progressed under immune-checkpoint inhibition therapy. To visualize the effects of the attributes on the class probabilities (response/progress under therapy), we generated a nomogram using a Naïve-Bayes classifier that was trained on the attributes of treatment results as described previously^24^. Statistical analysis was performed with Python (version 3.7, https://www.python.org/), R, the R Project (version 4.0.3, https://www.r-project.org/) and the statistical software package IBM SPSS (version 25.0). Statistical testing was carried out by using X^2^ test, Fisher’s test or Student's t-test. Survival rates were calculated by the Kaplan–Meier method and compared using log-rank. *p* < 0.05 was considered to be significant.

### Ethics approval

The study protocol conformed to the ethical guidelines of the 1975 Declaration of Helsinki and was approved by the Ethics Committee of the Medical Faculty of University of Cologne (Registration No. 08-144). Informed consent has been obtained.

## Data Availability

Data supporting the findings of this manuscript are available from the corresponding author upon reasonable request.

## Abbreviations

CNN: Convolutional neural network
CPI: Immune checkpoint inhibitor
CTLA-4: Cytotoxic T-lymphocyte-associated antigen 4
MM: Malignant melanoma
PD-1/PD-L1: Programmed death 1/programmed death-ligand 1
TIL: Tumor-infiltrating lymphocyte
